# SEARCH 8Es: A novel point of care ultrasound protocol for patients with chest pain, dyspnea or symptomatic hypotension in the emergency department

**DOI:** 10.1371/journal.pone.0174581

**Published:** 2017-03-29

**Authors:** Jung Hwan Ahn, Jin Jeon, Hong-Chuen Toh, Vicki Elizabeth Noble, Jun Su Kim, Young Sik Kim, Han Ho Do, Young Rock Ha

**Affiliations:** 1 Department of Emergency Medicine, Ajou University School of Medicine, Suwon, Republic of Korea; 2 Department of Emergency Medicine, Bundang Jesaeng General Hospital, Sungnam, Republic of Korea; 3 Acute and Emergency Care Centre, Khoo Teck Puat Hospital, Singapore, Singapore; 4 Department of Emergency Medicine, University Hospital-Cleveland Medical Center, Cleveland, Ohio, United States of America; 5 Department of Emergency Medicine, Dongguk University Ilsan Hospital, Graduate School of Medicine, Dongguk University, Goyang, Republic of Korea; University of Florida, UNITED STATES

## Abstract

**Objective:**

This study was conducted to evaluate a problem-oriented focused torso bedside ultrasound protocol termed “Sonographic Evaluation of Aetiology for Respiratory difficulty, Chest pain, and/or Hypotension” (SEARCH 8Es) for its ability to narrow differential diagnoses and increase physicians’ diagnostic confidence, and its diagnostic accuracy, for patients presenting with dyspnea, chest pain, or symptomatic hypotension.

**Methods:**

This single-center prospective observational study was conducted over 12 months in an emergency department and included 308 patients (184 men and 124 women; mean age, 67.7 ± 19.1 years) with emergent cardiopulmonary symptoms. The paired *t*-test was used to compare the number of differential diagnoses and physician’s level of confidence before and after SEARCH 8Es. The overall accuracy of the SEARCH 8Es protocol in differentiating 13 diagnostic entities was evaluated based on concordance (kappa coefficient) with the diagnosis made by the inpatient specialists. Sensitivity, specificity, positive predictive value, and negative predictive value were calculated.

**Results:**

SEARCH 8Es narrows the number of differential diagnoses (2.5 ± 1.5 vs. 1.4 ± 0.7; *p* < 0.001) and improves physicians’ diagnostic confidence (2.8 ± 0.8 vs. 4.3 ± 0.9; *p* < 0.001) significantly. The overall kappa coefficient value was 0.870 (*p* < 0.001), with the overall sensitivity, specificity, positive predictive value, and negative predictive value at 90.9%, 99.0%, 89.7%, and 99.0%, respectively.

**Conclusion:**

The SEARCH 8Es protocol helps emergency physicians to narrow the differential diagnoses, increase diagnostic confidence and provide accurate assessment of patients with dyspnea, chest pain, or symptomatic hypotension.

## Introduction

Focused bedside ultrasound is widely used by emergency physicians to evaluate critically ill patients. It could confirm the diagnosis or narrow the differentials suggested by history-taking and physical examination, and occasionally reveal critical conditions that are unsuspected on clinical grounds. This increased diagnostic capability aided the clinical decision making process, and allowed time sensitive intervention to be initiated [1–4].

Emergency physicians often encounter patients with complaints of chest pain, dyspnea or symptomatic hypotension. These complaints pose a real diagnostic dilemma, ranging from non-urgent diagnoses to life-threatening conditions requiring immediate recognition and interventions [5–7]. The presentations also varied, ranging from one to a combination of the three symptoms, depending on the severity of underlying disease process and the presence or absence of complications [8]. Formulating a diagnostic plan for these patients is a challenge for the physicians at the frontline. The incorporation of bedside ultrasound in the evaluation of such patients can help physicians to make more accurate and timely diagnoses [1, 9–16].

In 2010, we created a focused and integrated bedside ultrasound protocol for the systematic evaluation of patients presenting with acute respiratory distress, chest pain or symptomatic hypotension in emergency department. It is named ‘*Sonographic Evaluation of Aetiology of Respiratory difficulty*, *Chest pain and Hypotension using 8 E*’, abbreviated as ‘SEARCH 8Es’. The 8 *Es* refers to empty thorax, edematous lung, extended focused assessment with sonography for trauma (E-FAST), effusion, equality (the ratio between left and right ventricle), ejection fraction, exit (aorta) and entrance (inferior vena cava [IVC]) and endocardial movement.

We conducted this study to assess the efficacy of SEARCH 8Es with three goals in mind. Firstly, to evaluate if it narrows the differential diagnoses and secondly, if it increases the physicians’ level of confidence in the diagnosis. Lastly, to determine its accuracy and test characteristics.

## Materials and methods

### Study design and setting

This single-center, prospective, observational study was conducted in emergency department between January, 2011 and January, 2012 after obtaining the approval (EMC11-01) of the Institutional Review Board of Bundang Jesaeng General Hospital (Seongnam, Republic of Korea) with a waiver of informed consent.

### Eligibility

Inclusion criteria for the study were patients who are: (1) greater than 18 years of age, and with (2) a chief complaint of respiratory distress or chest pain, (3) hypotension defined as systolic blood pressure less than 90 mmHg or (4) signs and symptoms suggestive of shock regardless of systolic blood pressure (*e*.*g*., with cold clammy skin, confusion, agitation, altered mental status).

Exclusion criteria are patients: (1) in cardiac arrest, or (2) with critical diagnosis that can be made clinically and require immediate intervention (e.g. active gastrointestinal bleeding, known drug overdose), or (3) with a history of orthostatic hypotension and presented with symptomatic postural hypotension as the only chief complaint, or (4) with a history of low baseline blood pressure and not having any signs or symptoms related to hypotension, or (5) discharged or transferred to another hospital after initial emergency department (ED) evaluation.

A total of 352 eligible patients visited an emergency care center of our medical institution during the study period. Of these, 47 patients were subsequently excluded from the analysis: 15 refused to be admitted and 32 were referred from other hospitals with pre-determined diagnoses. Therefore, we enrolled a total of 308 patients in the current study.

### Diagnostic protocol

The SEARCH 8Es protocol is conceptualized to provide acute care physicians with a framework of utilizing cardiovascular ultrasound, lung ultrasound and focused assessment with sonography for trauma in a clinically integrated fashion. The existing literature on goal-directed ultrasound for evaluation of respiratory distress, chest pain or symptomatic hypotension was searched and reviewed. Conditions that can be diagnosed with bedside ultrasound are identified [1, 7, 17] and grouped under 8 goals, or 8Es. The scanning sequence, imaging views, key sonographic findings and diagnostic implication are then integrated in a practical and clinically meaningful manner.

**Fig 1:**
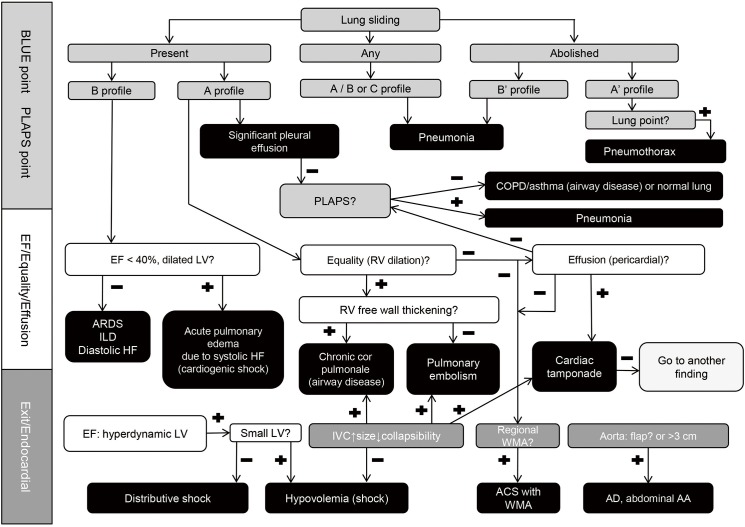
The algorithm for SEARCH 8Es. *Profiles*: A, lung sliding and A lines in both the lungs; A’, A profile without lung sliding and lung point; B, anterior-predominant bilateral B lines with lung sliding; B’, B profile without lung sliding; A/B, anterior-predominant B lines in one lung and predominant A lines in the other; C, anterior alveolar consolidation(s). *Abbreviations*: AAA, abdominal aortic aneurysm; ACS, acute coronary syndrome; AD, aortic dissection; ARDS, acute respiratory distress syndrome; BLUE, bedside lung ultrasound in emergency; COPD, chronic obstructive pulmonary disease; EF, ejection fraction; HF, heart failure; ILD, interstitial lung disease; IVC, inferior vena cava; LV, left ventricle; PLAPS, posterolateral alveolar/or pleural syndrome; RV, right ventricle; SEARCH, sonographic evaluation of aetiology for respiratory difficulty, chest pain, and/or hypotension; WMA, wall motion abnormality.

Twelve critical disease entities were categorized based on SEARCH 8Es. Table 1 shows the 8Es, disease entities and key sonographic findings associated with the 8Es. The integrated algorithm is depicted in Fig 1. As shown in Table 1 and Fig 1, the 12 categories are as follow: (1) Pulmonary embolism, (2) Airway disease (Asthma and chronic obstructive pulmonary disease [COPD]), (3) Pneumothorax, (4) Large pleural effusion due to other systemic disease (Liver cirrhosis and end-stage renal disease [ESRD]), (5) Pneumonia, (6) Acute pulmonary edema due to systolic heart failure, (7) Acute respiratory distress syndrome (ARDS), interstitial lung disease (ILD) or acute pulmonary edema due to diastolic heart failure, (8) Pericardial effusion (including cardiac tamponade), (9) Acute coronary syndrome with regional wall motion abnormality (ACS with RWMA), (10) Aortic aneurysm (AA) / Aortic dissection (AD), (11) Hypovolemic shock, (12) Septic shock.

**Table 1:** Sonographic findings in each category of the SEARCH 8Es protocol.

Disease entity	Eight goals							
	Lung, diaphragmatic ultrasound and E-FAST			Cardiovascular ultrasound				
	Empty thorax	Edematous or wet lung	E-FAST	Effusion (pericardial)	Equality (RV dysfunction)	EF (LV dysfunction)	Exit and entrance	Endocardial movement
Inconclusive	Nude profile			None or normal				
(Tension) Pneumothorax	no B profile + A’ profile ± lung point			Possibly displaced heart to the unaffected side, if it is large				
				Normal	Normal	Normal	Normal or dilated IVC (tension pneumothorax)	Normal
Acute pulmonary edema due to systolic dysfunction (±cardiogenic shock)	B profile ± pleural effusion			Normal	Normal	Low EF + usually dilated LV/LA	±dilated IVC	±extensive WMA (AMI)
ARDS, ILD, pulmonary edema due to diastolic HF	B profile ± other pneumonia profile (see also pneumonia, ARDS)			Normal	Normal or possible RV enlargement (chronic ILD or ARDS)	Normal or high EF	±dilated IVC	Normal
Airway disease (COPD, asthma)	Nude profile			Normal	Normal or possible RV enlargement (cor pulmonale)	Normal	±dilated IVC	Normal
Pulmonary embolism	Nude profile, ±PLAPS pattern			Normal	Positive RV enlargement	Normal	Dilated IVC	Paradoxical septal motion
Pneumonia	A profile + PLAPS pattern; A/B, B’, or C profile			Possibly displaced heart to the affected side, if it is atelectasis				
				Normal	Normal	Normal or high EF	Normal	Normal
Significant pleural effusion	Much pleural effusion + atelectasis ± peritoneal effusion			Possibly displaced heart to the unaffected side				
				+/-	Normal	Normal	±small IVC (trauma)	Normal
Cardiac tamponade	Nude profile			+	Diastolic RV collapse	Normal or high EF	Dilated IVC	Normal
ACS with regional WMA	Nude profile, possible B profile (cardiogenic shock)			Possible (free wall rupture)	Possible (RV infarct)	Usually low EF	Possible MR (PM rupture)	positive
AA, AD	Nude profile ± peritoneal effusion (ruptured acute AA)			Possible (AD)	Normal	Normal	Aneurysm (>3 cm) or flap, possible AR (AD)	Normal
Hypovolemia (±shock)	Nude profile (+PLAPS pattern in thoracic trauma with hemothorax ± lung contusion)			Normal	Normal	Small hyperkinetic LV	Small IVC	Normal
Vasodilation (distributive shock)	Nude profile			Normal	Normal	Normal-size hyperkinetic LV	±small IVC	Normal

*Profiles*: A, lung sliding and A lines in both the lungs; A’, A profile without lung sliding and lung point; B, anterior-predominant bilateral B lines with lung sliding; B’, B profile without lung sliding; A/B, anterior-predominant B lines in one lung and predominant A lines in the other; C, anterior alveolar consolidation(s). *Abbreviations*: AA, aortic aneurysm; ACS, acute coronary syndrome; AD, aortic dissection; AMI, acute myocardial infarction; AR, aortic regurgitation; ARDS, acute respiratory distress syndrome; COPD, chronic obstructive pulmonary disease; EF, ejection fraction; E-FAST, extended focused assessment with sonography for trauma; HF, heart failure; ILD, interstitial lung disease; IVC, inferior vena cava; LA, left atrium; LV, left ventricle; MR, mitral regurgitation; PLAPS, posterolateral alveolar/pleural syndrome; PM, papillary muscle; RV, right ventricle; SEARCH, sonographic evaluation of aetiology for respiratory difficulty, chest pain, and/or hypotension; WMA, wall motion abnormality

Therefore, the SEARCH 8Es protocol covers twelve critical diagnoses. The thirteenth entity includes patients with diagnoses outside the twelve or in whom ultrasound findings were indeterminate.

### Ultrasound-assisted diagnosis

**Fig 2:**
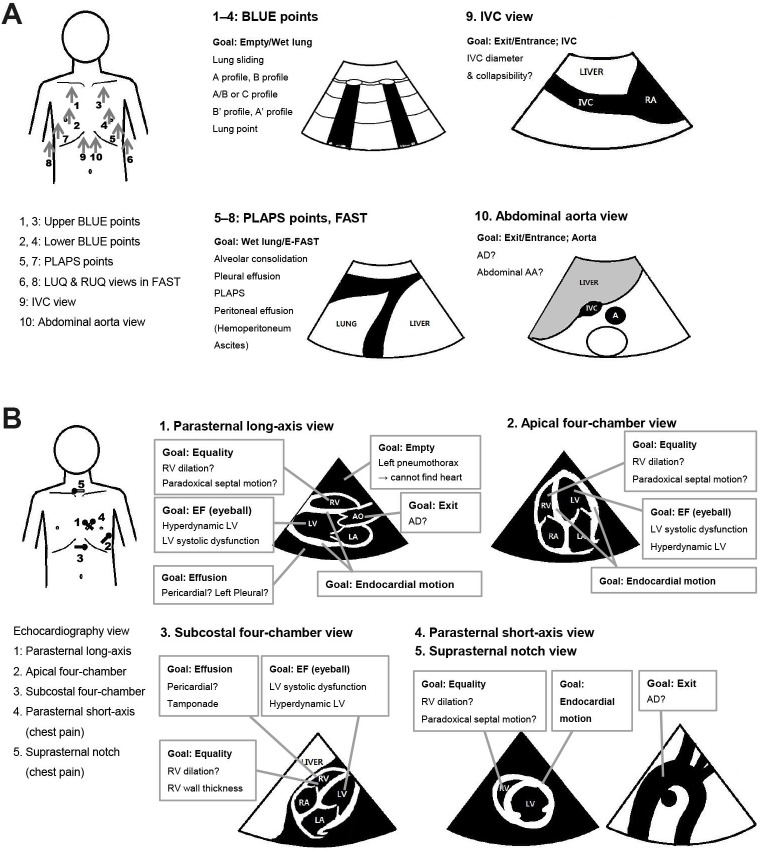
Schematic drawing of transducer, the image planes and key points in the SEARCH 8Es protocol. The performance sequence of SEARCH 8Es was divided into two steps. (A) The first step of ‘SEARCH’ with the convex probe to search for the pneumothorax, interstitial syndrome (pulmonary edema, ARDS, interstitial disease), pneumonia, pleural effusion, peritoneal effusion, abdominal aortic aneurysm, aortic dissection, or a clue of hypovolemia. The convex probe is located longitudinally on the anterior chest and posterolateral chest to examine whether there are any findings that are suggestive of empty thorax, edematous or wet lung and free fluid above and below the diaphragm. In cases of intraperitoneal fluid, the probe is placed in the same manner as the conventional FAST. The convex probe is used to evaluate the lungs, the abdomen and to look at the IVC. The A profile is defined the conditions appeared lung sliding with A line in both lungs. The A’ profile is an A profile without lung sliding and lung point. The B profile is defined to anterior-predominant bilateral B lines associated with lung sliding. The B’ profile is a B profile without lung sliding. The A/B profile is defined to anterior predominant B lines on one lung and predominant A lines on the other. The C profile is defined to anterior alveolar consolidation(s). (B) The 2^nd^ step of ‘SEARCH’ with the cardiac probe to search for pericardial effusion with or without tamponade physiology (diastolic right ventricle collapse), pulmonary embolism (right ventricle enlargement, paradoxical interventricular septal movement), left ventricle systolic dysfunction, acute myocardial infarction (left and right ventricular regional wall motion abnormality) and it’s mechanical complications (papillary muscular rupture, left ventricle wall rupture), and a clue of a hypovolemic or distributive shock (hypokinetic small-sized left ventricle or hyperkinetic normal-sized left ventricle). In case of chest pain, parasternal short axis view and suprasternal notch view was performed due to the evaluation of regional wall motion and thoracic aorta dissection or aneurysm. If the apical 4 chamber view is difficult due to poor window, a subxiphoid view is used. AAA, abdominal aortic aneurysm; AD, aortic dissection; ARDS, acute respiratory distress syndrome; BLUE, bedside lung ultrasound in emergency; EF, ejection fraction; E-FAST, extended focused assessment with sonography for trauma; FAST, focused assessment with sonography for trauma; IVC, inferior vena cava; LA, left atrium; LUQ, left upper quadrant; LV, left ventricle; PLAPS, posterolateral alveolar and/or pleural syndrome; RA, right atrium; RUQ, right upper quadrant; RV, right ventricle; SEARCH, sonographic evaluation of aetiology for respiratory difficulty, chest pain, and/or hypotension.

The SEARCH 8Es protocol was performed sequentially in two steps. Fig 2A and 2B show the probe locations, imaging view and goals, and key findings in each step. If the apical 4 chamber window is difficult obtain in step 2, the subxiphoid view is used instead. The size of the chambers, ejection fraction and severity of RWMA (if any) are estimated [18].

A 6–2 MHz convex probe and a 4–2 MHz cardiac probe (ACUSON X500, Siemens Medical solutions, Malvern, PA, USA) were used. The physicians performing the scans were a board-certified emergency physician and 2 senior emergency medicine residents. All three physicians have experience performing bedside ultrasound in abdominal, cardiovascular and lung applications. The board-certified emergency physician is a faculty in WINFOCUS group (World Interactive Network Focused on Critical Ultrasound group) and has the experience of abdominal ultrasound for 10 years, echocardiography for 8 years, and lung ultrasound for 5 years. The two senior residents have received a hands-on training for SEARCH 8E’s every two weeks for more than one year by the board-certified emergency physician.

### Evaluation and criteria

The number of differential diagnoses and the physician’s level of confidence in diagnosis were assessed both before and after applying the SEARCH 8Es protocol. The emergency physician selects the differentials from the predefined list of 13 disease entities (12 specific diagnoses and 1 others category). A 5-point Likert scale was used to assess confidence level, with the highest confidence level assigned 5 points and the lowest level given 1 point. Upon completion of the SEARCH 8Es protocol, the emergency physician identified a primary diagnosis from the list of thirteen which best account for the patients’ presentation to the ED.

The criterion standard was determined by the inpatient specialists, who were blinded to the SEARCH 8Es findings. After an enrolled patient was discharged, the treating inpatient specialist was asked to select one primary diagnosis, from the same list of thirteen disease entities, which best account for the patient’s initial ED presentation. This diagnosis is the criterion standard with which the accuracy of SEARCH 8Es is evaluated.

### Statistical analysis

All data were analyzed by using SPSS 19 statistics software (IBM, Inc., Armonk, NY, USA). Continuous data are expressed as means (standard deviation). Categorical data are shown as absolute values, together with the frequency distribution where appropriate.

The paired *t*-test was used to compare the number of impressions and level of confidence before and after applying the SEARCH 8Es protocol.

The concordance (kappa coefficient) between the final discharge diagnosis and the primary diagnosis derived from the SEARCH 8Es performed in the ED was examined to determine the overall accuracy. Sensitivity, specificity, positive predictive value (PPV), and negative predictive value (NPV) were measured to ascertain the diagnostic performance. A *p*-value less than 0.05 was considered significant.

A sample size of 216 achieves 80% power to detect a difference of -0.07 between the null hypothesis correlation of 0.76 and the alternative hypothesis correlation of 0.83 using a two-sided hypothesis test with a significance level of 0.05 by PASS 12 (NCSS, LLC. Kaysville, Utah, USA). The null hypothesis correlation of 0.76 and the alternative hypothesis correlation of 0.83 were chosen by prior study [10]. Therefore, the total number of 308 patients enrolled in the current study was sufficient to achieve the power of this current study. However, there was the difference between the prior study and this current study. Therefore, the power analysis was conducted to confirm the validity of sample size in this current study by using PASS 12 (NCSS, LLC. Kaysville, Utah, USA) whether the sample size achieved the sufficient power for our three goals of this study: (1) narrows the differential diagnoses. (2) increases the physicians’ level of confidence in the diagnosis (3) determines its accuracy and test characteristics (kappa coefficient).

## Results

The number of differential diagnoses was significantly reduced from 2.5 to 1.4 (2.5 ± 1.5 vs. 1.4 ± 0.7; *p* < 0.001) after SEARCH 8Es was performed. The level of confidence in the diagnosis was also increased significantly (2.8 ± 0.8 vs. 4.3 ± 0.9; *p* < 0.001). For diagnostic accuracy, the overall concordance rate with the criterion standard was 89.0% (274/308), with an overall kappa coefficient value of 0.870 (*p* < 0.001). Table 2 shows the kappa coefficient value, sensitivity, specificity, PPV, and NPV according to the 13 diagnostic entities covered in the SEARCH 8Es protocol.

**Table 2:** Accuracy data of the SEARCH 8Es protocol for the 13 target disease entities (%).

Disease entity	Sensitivity	Specificity	PPV	NPV	Kappa	*p*
Inconclusive	73.8 (31/42)	97.7 (260/266)	83.8 (31/37)	95.9 (260/271)	0.753	<0.001
Pneumothorax	100 (16/16)	100 (292/292)	100 (16/16)	100 (292/292)	1.000	<0.001
Acute pulmonary edema	94.3 (66/70)	97.9 (233/238)	93.0 (66/71)	98.3 (233/237)	0.917	<0.001
ARDS, chronic ILD, or diastolic HF	100 (4/4)	100 (304/304)	100 (4/4)	100 (304/304)	1.000	<0.001
Airway disease (COPD, asthma)	76.5 (13/17)	98.6 (287/291)	76.5 (13/17)	98.6 (287/291)	0.751	<0.001
Pulmonary embolism	90.9 (10/11)	100 (297/297)	100 (10/10)	99.7 (297/298)	0.951	<0.001
Pneumonia	90.9 (40/44)	97.7 (258/264)	87.0 (40/46)	98.5 (258/262)	0.870	<0.001
Significant pleural effusion	100 (14/14)	99.3 (292/294)	87.5 (14/16)	100 (292/292)	0.930	<0.001
Pericardial effusion or cardiac tamponade	100 (2/2)	100 (306/306)	100 (2/2)	100 (306/306)	1.000	<0.001
ACS with WMA	91.2 (62/68)	97.6 (235/240)	92.5 (62/67)	97.6 (235/241)	0.896	<0.001
AD or AA	100 (7/7)	100 (301/301)	100 (7/7)	100 (301/301)	1.000	<0.001
Hypovolemic shock	100 (2/2)	98.4 (301/306)	28.6 (2/7)	100 (301/301)	0.439	<0.001
Sepsis (distributive shock)	63.6 (7/11)	99.7 (296/297)	87.5 (7/8)	98.7 (296/300)	0.729	<0.001
Total	90.9	99.0	89.7	99.0	0.870	<0.001

AA, aortic aneurysm; ACS, acute coronary syndrome; AD, aortic dissection; ARDS, acute respiratory distress syndrome; COPD, chronic obstructive pulmonary disease; HF, heart failure; ILD, interstitial lung disease; NPV, negative predictive value; PPV, positive predictive value; SEARCH, sonographic evaluation of aetiology for respiratory difficulty, chest pain, and/or hypotension; WMA, wall motion abnormality

Table 3 shows the patients characteristics in terms of the final diagnoses and chief complaints. One hundred and eighty-six patients (60.4%) had only one symptom and 122 patients (39.6%) had 2 or more symptoms. 226 patients (86.4%) had a final diagnoses that can be identified by SEARCH 8Es (i.e. within the twelve diagnostic entities), with the remaining 42 patients (13.6%) falling beyond the scope of the protocol. The diagnoses of these 42 patients are also summarized in Table 3. Among 18 cases of ACS without RWMA, 14 cases (77.8%) were classified as inconclusive and 4 cases (12.2%) were misdiagnosed to be ACS with RWMA. One of 4 arrhythmia cases was misdiagnosed as ACS with RWMA. All 4 cases of gastrointestinal origin were judged inconclusive. One of three cases of other pulmonary disease was misdiagnosed as airway disease. All 4 ESRD cases with congestive HF were misdiagnosed as acute pulmonary edema due to congestive HF. Among 9 cases of nonspecific diagnosis, one case was misdiagnosed as airway disease.

**Table 3:** General patient characteristics in terms of the symptoms and final diagnoses (*n*, %).

Characteristic/Diagnosis	Dyspnea	Chest pain	Hypotension	Dyspnea + chest pain	Dyspnea + hypotension	Chest pain + hypotension	All symptoms	Total
Mean age (years)	73.5 ± 16.0	54.1 ± 18.7	70.3 ± 12.1	63.2 ± 21.4	75.7 ± 16.6	71.2 ± 15.3	68.2 ± 24.3	67.7 ± 19.1
Gender (male:female)	46:52	55:11	11:11	24:18	27:19	17:8	4:5	184:124
***Twelve disease entities in scope of SEARCH 8Es***								
Pneumothorax	1 (1.0)	5 (7.6)	0 (0)	9 (21.4)	0 (0)	0 (0)	1 (11.1)	16 (5.2)
Acute pulmonary edema	42 (42.9)	0 (0)	0 (0)	14 (33.3)	12 (26.1)	1 (4.0)	1 (11.1)	70 (22.7)
ARDS, chronic ILD, diastolic HF	2 (2.0)	0 (0)	1 (4.5)	0 (0)	1 (2.2)	0 (0)	0 (0)	4 (1.3)
Airway disease (COPD, asthma)	14 (14.3)	0 (0)	0 (0)	0 (0)	3 (6.5)	0 (0)	0 (0)	17 (5.5)
Pulmonary embolism	3 (3.1)	0 (0)	2 (9.1)	4 (9.5)	1 (2.2)	0 (0)	1 (11.1)	11 (3.6)
Pneumonia	19 (19.4)	0 (0)	3 (13.6)	3 (7.1)	15 (32.6)	2 (8.0)	2 (22.2)	44 (14.3)
Significant pleural effusion	8 (8.2)	0 (0)	1 (4.5)	1 (2.4)	4 (8.7)	0 (0)	0 (0)	14 (4.5)
Pericardial effusion	0 (0)	0 (0)	0 (0)	0 (0)	2 (4.3)	0 (0)	0 (0)	2 (0.6)
ACS with WMA in echocardiography	3 (3.1)	35 (53.0)	6 (27.3)	6 (14.3)	3 (6.5)	12 (48.0)	3 (33.3)	68 (22.1)
AD or AA	0 (0)	0 (0)	1 (4.5)	0 (0)	2 (4.3)	4 (16.0)	0 (0)	7 (2.3)
Hypovolemic shock	0 (0)	0 (0)	2 (9.1)	0 (0)	0 (0)	0 (0)	0 (0)	2 (0.6)
Sepsis (distributive shock)	0 (0)	0 (0)	6 (27.3)	0 (0)	1 (2.2)	3 (12.0)	1 (11.1)	11 (3.6)
***The disease entities out of scope of SEARCH 8Es***								
ACS without WMA in echocardiography	0 (0)	14 (21.2)	0 (0)	2 (4.8)	0 (0)	2 (8.0)	0 (0)	18 (5.8)
Arrhythmia	0 (0)	1 (1.5)	0 (0)	0 (0)	2 (4.3)	1 (4.0)	0 (0)	4 (1.3)
Gastrointestinal origin	0 (0)	3 (4.5)	0 (0)	1 (2.4)	0 (0)	0 (0)	0 (0)	4 (1.3)
Other pulmonary disease	2 (2.0)	1 (1.5)	0 (0)	0 (0)	0 (0)	0 (0)	0 (0)	3 (1.0)
ESRD	3 (3.1)	0 (0)	0 (0)	1 (2.4)	0 (0)	0 (0)	0 (0)	4 (1.3)
Nonspecific diagnosis	1 (1.0)	7 (10.6)	0 (0)	1 (2.4)	0 (0)	0 (0)	0 (0)	9 (2.9)
Total	98 (31.8)	66 (21.4)	22 (7.1)	42 (13.6)	46 (14.9)	25 (8.1)	9 (2.9)	308 (100)

AA, aortic aneurysm; ACS, acute coronary syndrome; AD, aortic dissection; ARDS, acute respiratory distress syndrome; COPD, chronic obstructive pulmonary disease; ESRD, end-stage renal disease; HF, heart failure; ILD, interstitial lung disease; SEARCH, sonographic evaluation of aetiology for respiratory difficulty, chest pain, and/or hypotension; WMA, wall motion abnormality

A sample size of 308 achieves 100% power to detect a mean of paired differences (a known standard deviation of differences), 1.1 (1.4) in differential diagnosis and 1.4 (0.9) in the level of confidence, and with a significance level (alpha) of 0.05 using a two-sided paired z-test. Moreover, a test for agreement using the Kappa statistic revealed that 100% power was achieved by using a difference of -0.11 between the null hypothesis correlation of 0.76 and the alternative hypothesis correlation of 0.87 using a two-sided hypothesis test with a significance level of 0.05. The data in the table is the minimal data set underlying the findings of the study (S1 Table.).

## Discussion

In the management of the undifferentiated critically ill patient, emergency physicians are constantly challenged to narrow the diagnostic conundrum rapidly and effectively at the bedside. The use of point of care ultrasound has positively influenced and speed up this process. Several problem-oriented focused bedside ultrasound scanning protocols have been proposed in the field of critical care and emergency medicine [1, 2, 10, 13, 14, 16, 19, 20]. Lichtenstein and Meziere [1] describe the BLUE protocol for patients with acute respiratory failure. Copetti et al. [16, 20] have added cardiac ultrasound to the lung ultrasound protocols to rule out and define other diseases in patients with dyspnea and “dry” lung patterns (A-profile) of non-cardiac origin on lung ultrasound. Bataille et al. [12] and Kajimoto et al. [14] have reported better performance of the integrated use of bedside lung ultrasound and echocardiography than lung ultrasound alone in the diagnosis of acute respiratory failure. Most recent studies have reported that the effectiveness of bedside multi-organ point-of-care ultrasound in undifferentiated shock by using IVC size and respiratory variation as an indicator for fluid resuscitation [9–11, 13, 15]. Using information from lung and IVC ultrasound, physician can embark on a treatment plan, and be guided subsequently by cardiac ultrasound [21]. Most ultrasound guided resuscitation protocols are designed to evaluate patients with hypotension or dyspnea using basic cardiovascular and lung ultrasound findings [1, 2, 10, 13, 14, 16, 19, 20]. As many acute care providers have gained the required competency, it is opportune to explore the expansion of the armamentarium that point of care ultrasound can provide. Patients with chest pain, in particular, should prompt closer evaluation for ACS, pericarditis, or aortic dissection [22]. The SEARCH 8Es is a more advanced resuscitation ultrasound protocol developed to fulfil this need at the bedside. This study has demonstrated its effectiveness in reducing the physicians’ diagnostic uncertainty, as well as its accuracy in diagnosing most of the life-threatening cardiopulmonary conditions. Employing the spatial contiguity principle [23], with illustrative images sited adjacent to clear and succinct instructions, the SEARCH 8Es handouts can be easily understood and followed through. The organization of the scanning sequence and sonographic evaluation into 8 distinct goals also facilitates training in this protocol. In fact, the components of the 8Es can serve as individual instructional tools for clinicians learning resuscitation ultrasound, culminating in the integrated algorithm presented in this paper.

As compared with previous reports, our results showed a relatively higher *κ*-coefficient, sensitivity, specificity, PPV and NPV for all 11 categories except for airway disease and septic shock [1, 6, 12, 16, 17, 24]. One explanation for the greater accuracy in these eleven categories is that physicians were not blinded to patient characteristics and clinical information. Nevertheless, these results retain their significance as goal-directed and focused bedside ultrasound, such as SEARCH 8Es, is regarded as an extension of physical examination. The blinding of ultrasound findings would add little value to clinical practice as these sonographic findings are integrated with physical evaluation in the real world of clinician performed ultrasound, and not interpreted in isolation [1–3, 16, 20, 25, 26]. On the other hand, point of care ultrasound could be used in conjunction with other history and physical exam findings and a physicians’ clinical experience might be an essential factor for the efficiency of SEARCH 8Es in a clinical setting. Possible reasons for the less than expected performance in patients with airway disease and sepsis include: (1) a relatively small number of patients with airway disease, hypovolemic shock or septic shock (n = 30), in which the study was underpowered to detect any clinically significant difference (2) Airway diseases, such as asthma or COPD, may require more extensive lung evaluations than those that were mandated by the SEARCH 8Es protocol. In the current study, there were four incorrect cases of positive posterolateral alveolar/pleural syndrome (PLAPS) in patients with COPD who ultimately did not have pneumonia. In addition, SEARCH identified three patients with airway disease and normal lung patterns. They were subsequently found to have abnormal pulmonary functions after admission.

It is also notable that in many cases of septic shock, it is difficult to distinguish between septic shock and dehydration from other causes as their presenting signs and symptoms overlapped. In our current study, a hyper-dynamic left ventricle was seen in four misdiagnosed cases of septic shock without fever, which again points to this as a very important finding for patients presenting to the ED with shock. On the other hand, volume responsiveness may be an equally important diagnostic consideration for the treating physicians, rather than simply differentiating between sepsis and hypovolemia. A Doppler study can provide estimation of volume responsiveness (velocity time integral in apical 5 chamber view or carotid view) and the systemic vascular resistance. Further studies are warranted to examine the usefulness of SEARCH 8Es combined with a Doppler study in differentiating between and managing patients with septic and hypovolemic shock.

The patients in whom SEARCH 8Es resulted in misdiagnoses were analyzed. (1) The most common misdiagnoses were associated with RWMA. Of the 86 cases of ACS with (n = 68) or without RWMA (n = 18), nine were misdiagnosed. This arises mainly from differentiating the subtle differences between normal- and abnormal-segment motions and difficulty in obtaining optimal imaging conditions [27, 28]. Some patients had suboptimal views as they could not be rotated to improve the acoustic window. The presence of pre-existing RWMA also confounded the evaluation. Indeed, it was also previously reported that RWMA on a 2-D echocardiography posed a diagnostic dilemma even for cardiologists [27, 28]. Therefore, it is more important to recognize the limitations of RWMA in the assessment of patients presenting with possible ACS and take into account the contexual and intrinsic limitations of RWMA when applying the SEARCH 8Es. (2) In patients with 2 or more concurrent conditions, particularly those with underlying cardiopulmonary diseases, SEARCH 8Es may not produce conclusive categorization of diagnoses. These complex cases were difficult to diagnose and manage, even for the inpatient specialists. In this study, there were four incorrect cases among five patients with either pre-existing COPD or congestive HF. In two patients with COPD without pulmonary edema, SEARCH 8Es’ revealed positive PLAPS without B-profile, and therefore, given the initial diagnosis of pneumonia. However, the final diagnosis was revised to pleural effusion and COPD. In one cases with lung cancer and old myocardial infarction, ACS with RMWA was strongly suspected. However, finial diagnosis was lung cancer. In one case with congestive HF and COPD, SEARCH 8Es’ revealed both PLAPS and B-profile, therefore, making it difficult to distinguish between pneumonia versus pulmonary edema. The ED physician’s impression was pulmonary edema; while the final diagnosis was pneumonia with pleural effusion.

The study has these limitations. (1) A convenience sample was recruited, as the SEARCH 8Es was performed only when at least one of the three physicians trained this protocol was on duty. In addition, it is performed as a single emergency center. These factors limit its generalizability. (2) Patients who are not admitted or are transferred to other hospitals after the initial ED evaluation are excluded from the study in view of the difficulty in determining the criterion standard diagnosis. This could introduce a selection bias. (3) While the disease profiles of the 308 patients reflect the frequency of diseases encountered, the numbers for certain specific diseases, such as pericardial effusion, are small. Although it has been shown that an integrated ultrasound produced a high degree of accuracy in making a diagnosis of specific disease entities [1, 10, 13], the utility of SEARCH 8Es in this area could be further researched in larger-scale studies. (4) As the physician performing the initial clinical evaluation and subsequent SEARCH 8Es are the same person, the prior clinical impression could have influenced the way sonographic findings are interpreted. Nevertheless, SEARCH 8Es is designed as a sonographic framework that is integrated with, rather than apart from, clinical reasoning. Moreover, there are occasions where the bedside ultrasound revealed unexpected or even contradictory findings. This underscore the importance of sound clinical reasoning and expertise when interpreting a diagnostic tool to aid the clinical decision making process. (5) As SEARCH 8Es is performed by experienced providers in the research, the transferability of the protocol in emergency medicine setting would therefore depend on the contextual factors in which ultrasound is used in the various institutions, including the point of care ultrasound training for and experience of the providers. Unfortunately, in this study, the methods of education of SEARCH 8Es and practice duration in which the emergency physicians needed to apply in daily routine were not suggested. However, we believe that one year experiences might be required to conduct reliable SEARCH 8Es examination although there was no evidence.

In conclusion, the SEARCH 8Es protocol is an effective tool to help emergency physicians to narrows the differential diagnosis, increase diagnostic confidence and is accurate in the evaluation of patients with dyspnea, chest pain, or symptomatic hypotension. This research contributes to the body of evidence supporting the use of point of care ultrasound in the resuscitation of the critically ill.

## Supporting information

S1 Table Minimal data set underlying the findings of the study. (XLS)

## Abbreviations

AA: aortic aneurysm
ACS: acute coronary syndrome
AD: aortic dissection
AMI: acute myocardial infarction
AR: aortic regurgitation
ARDS: acute respiratory distress syndrome
BLUE: bedside lung ultrasound in emergency
COPD: chronic obstructive pulmonary disease
EF: ejection fraction
ESRD: end-stage renal disease
FAST: focused assessment with sonography for trauma
HF: heart failure
ILD: interstitial lung disease
IVC: inferior vena cava
LA: left atrium
LUQ: left upper quadrant
LV: left ventricle
MR: mitral regurgitation
NPV: negative predictive value
PLAPS: posterolateral alveolar/pleural syndrome
PM: papillary muscle
PPV: positive predictive value
RA: right atrium
RUQ: right upper quadrant
RV: right ventricle
SEARCH: sonographic evaluation of aetiology for respiratory difficulty, chest pain, and/or hypotension
WMA: wall motion abnormality
